# Cloning, Characterization and Anion Inhibition Studies of a β-Carbonic Anhydrase from the Pathogenic Protozoan *Entamoeba histolytica*

**DOI:** 10.3390/molecules23123112

**Published:** 2018-11-28

**Authors:** Susanna Haapanen, Silvia Bua, Marianne Kuuslahti, Seppo Parkkila, Claudiu T. Supuran

**Affiliations:** 1 Faculty of Medicine and Health Technology, Tampere University, 33100 Tampere, Finland; Haapanen.Susanna.E@student.uta.fi (S.H.); Marianne.Kuuslahti@staff.uta.fi (M.K.); seppo.parkkila@staff.uta.fi (S.P.); 2 Neurofarba Dept., Sezione di Scienze Farmaceutiche e Nutraceutiche, Università degli Studi di Firenze, Via U. Schiff 6, Sesto Fiorentino, 50019 Florence, Italy; silvia.bua@unifi.it; 3 Fimlab Ltd., Tampere University Hospital, 33100 Tampere, Finland

**Keywords:** carbonic anhydrase, metalloenzymes, protozoan, *Entamoeba histolytica*, anions, inhibitor

## Abstract

We report the cloning and catalytic activity of a β-carbonic anhydrase (CA, EC 4.2.1.1), isolated from the pathogenic protozoan *Entamoeba histolytica*, EhiCA. This enzyme has a high catalytic activity for the physiologic CO_2_ hydration reaction, with a k_cat_ of 6.7 × 10^5^ s^−1^ and a k_cat_/K_m_ of 8.9 × 10^7^ M^−1^ × s^−1^. An anion inhibition study of EhiCA with inorganic/organic anions and small molecules revealed that fluoride, chloride, cyanide, azide, pyrodiphosphate, perchlorate, tetrafluoroborate and sulfamic acid did not inhibit the enzyme activity, whereas pseudohalides (cyanate and thiocyanate), bicarbonate, nitrate, nitrite, diethyldithiocarbamate, and many complex inorganic anions showed inhibition in the millimolar range (K_I_s of 0.51–8.4 mM). The best EhiCA inhibitors were fluorosulfonate, sulfamide, phenylboronic acid and phenylarsonic acid (K_I_s in the range of 28–86 μM). Since β-CAs are not present in vertebrates, the present study may be useful for detecting lead compounds for the design of effective enzyme inhibitors, with potential to develop anti-infectives with alternative mechanisms of action.

## 1. Introduction

The carbonic anhydrases (CAs, EC 4.2.1.1) are enzymes that effectively catalyze the reaction between CO_2_ and water, yielding bicarbonate (HCO_3_^−^) and protons (H^+^). They are among the fastest catalysts known in nature [1,2,3]. CAs are multifunctional enzymes, which play a central role in different physiological, biochemical, and metabolic processes, such as acid-base homeostasis; respiratory gas exchange; electrolyte secretion; and biosynthesis of urea, glucose, fatty acids, and carbamoyl phosphate. They are also vital in ionic transport, muscular contraction (in vertebrates), and photosynthesis (in plants and algae). Seven distinct genetic families (i.e., the α, β, γ, δ, ζ, η, and θ class CAs) are known to date, with a wide distribution in organisms throughout the tree of life [4,5,6,7,8,9,10]. The CA classes do not share any significant sequence and structural identity, being a paradigmatic example of convergent evolution at the molecular level [1,2,3].

The first β-CA was discovered in 1939, but it took several decades until it was recognized as evolutionarily and structurally distinct from the previously studied CAs, those belonging to the α-class [11]. After the 1990s, many new β-CAs were discovered in the genomes of various organisms [11]. Based on current knowledge, these enzymes are found in photosynthetic organisms, eubacteria, yeasts, and Archaea [11,12,13]. Later on, it was discovered that they are also present in the genomes of insects, nematodes, and protozoans, but not in mammals [14]. Therefore, β-CAs are considered promising target enzymes for antiparasitic drugs [15,16,17]. The physiological significance of β-CAs is somehow ambiguous in most organisms studied so far, because frequently there are more than one enzyme class present [1,2,3,14,15,16,17,18]. However, β-CAs were shown to have important roles (for instance, in providing bicarbonate/CO_2_ for the photosynthetic enzyme Rubisco in the chloroplasts of many plants/algae [11,17,18]). *Helicobacter pylori* contains only one α- and one β-CA, whose inhibition with sulfonamides impairs the growth of the pathogen in vitro and in vivo [13,15]. The physiological relevance of β-CAs in many organisms, including protozoans belonging to the Amoebozoas, is yet to be discovered. However, in *Leishmania* spp., a β-class CA enzyme (LdcCA) was recently shown to be a potential drug target [19,20,21]. Indeed, the inhibition of protozoan β-CAs with sulfonamides, formulated as nanoemulsions, had a profound effect on the survival and growth of *L. amazonensis* and *L. infantum*, two species which provoke serious disease in the tropical and subtropical countries [21].

*Entamoeba histolytica* is a pathogenic protozoan human parasite causing amebiasis, which can be expressed as colitis or abscess of intestines or liver [22,23]. The common symptoms are diarrhea, colitis, and dysentery, but the majority of infections are asymptomatic [22,23]. *E. histolytica* is ingested with contaminated food or water as mature cysts, which excystate in the small intestine. The released trophozoites will then invade the large intestine [22]. *E. histolytica* is capable of lysing human tissues, killing immune effector cells by contact-dependent cytolysis [22,24]. The parasite has many virulence mechanisms, as it can adhere to host cells with multi-subunit GalGalNAc lectins, degrade the host extracellular matrix with cysteine proteases, and lyses target cells with amoebapores [25]. The invasive forms of the infection generally include cyst formation in the liver, which can lead to complications such as pleural effusion, due to the rupture of the cyst [22,26]. Rarely, they also disseminate through other extraintestinal organs (e.g., the brain or pericardium) [22,23]. Although there are effective medications for treating *E. histolytica*, therapies for the invasive forms produce many adverse side effects [22,23], and there are additional limitations to such therapies, among which is an increasing prevalence of resistance to commonly used drugs, which emphasizes the need for new drug targets against this protozoan [23,25]. Thus, we decided to clone and investigate in detail the β-CA present in this pathogenic protozoan. Here we report the cloning, purification, investigation of the catalytic activity, and the anion inhibition profile of the recombinant enzyme belonging to the β-class, identified in the genome of the pathogenic protozoan *E. histolytica*, denominated EhiCA.

## 2. Results and Discussion

**Figure 1:**
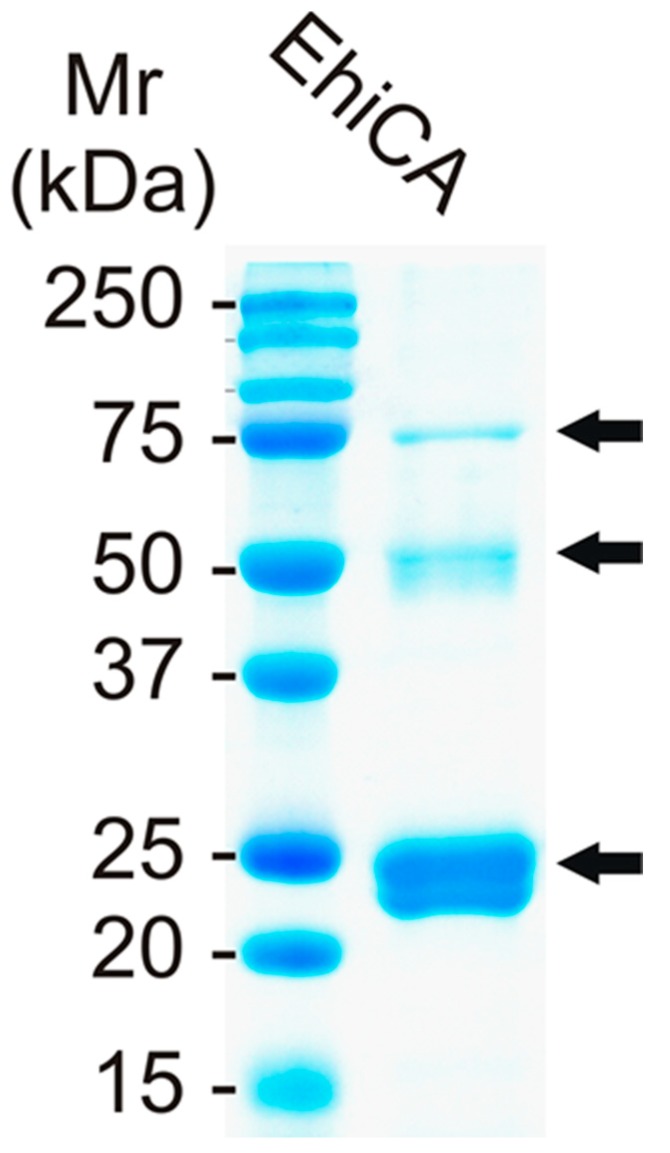
SDS PAGE of the β-CA from E. histolytica (EhiCA) showing a 21/25 kDa doublet polypeptide and additional polypeptides of about 50 and 75 kDa (arrows). Left lane: Standard Mw markers. The dimer and the trimer of EhiCA are also seen (arrows), as reported for other β-CAs cloned and purified earlier [11,12,13,14,15].

We produced the β-CA of *E. histolytica,* EhiCA, in the *E. coli* production system (see Experimental for details) as reported earlier for other CAs, such as hCA VII [19]. As a result, we obtained a 21 kDa protein, which was confirmed to be the right β-CA with mass spectrometry (MS) and SDS-PAGE (Figure 1). Furthermore, atomic absorption spectroscopy allowed us to determine the presence of one zinc ion per polypeptide chain (data not shown), which confirmed the MS data.

We measured the catalytic activity of the recombinant EhiCA (for the CO_2_ hydration reaction) [27], comparing its kinetic parameters with those of other such enzymes, belonging to the α-class, such as hCA I and II, (h stands for human isoform). Table 1 shows that EhiCA has a significant catalytic activity (for the physiologic reaction, CO_2_ hydration to bicarbonate and protons), with a k_cat_ of 6.7 × 10^5^ s^−1^ and a k_cat_/K_m_ of 8.9 × 10^7^ M^−1^ × s^−1^, being thus 1.8 times more effective as a catalyst, compared to the slow human isoform hCA I (considering the k_cat_/K_m_ values). Furthermore, like most enzymes belonging to the CA superfamily, EhiCA was inhibited by acetazolamide (AZA, 5-acetamido-1,3,4-thiadiazole-2-sulfonamide): A standard, clinically used sulfonamide CA inhibitor [1,2,3]. It can be observed that, similar to hCA I, EhiCA was inhibited in the high nanomolar range by this compound, with an inhibition constant K_I_s of 509 nM (Table 1).

**Figure 2:**
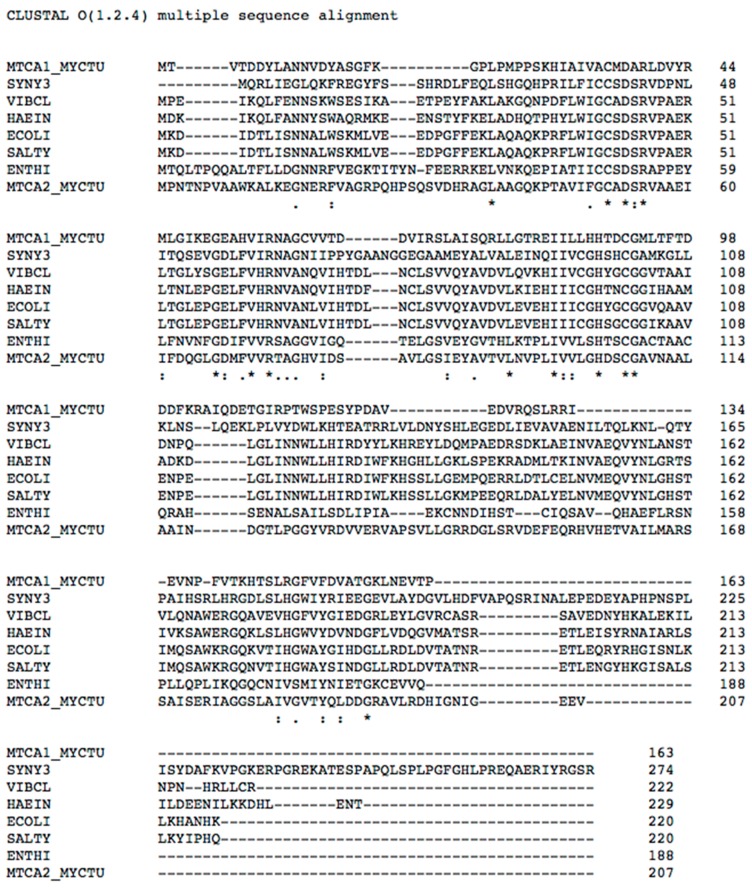
Multi-alignment of the amino acid sequences of the β-CAs from *M. tuberculosis* (isoform MTCA1_MYCTU), *Synechocystis* sp. (SYNY3), *V. cholerae* (VIBCL), *H. influenzae* (HAEIN), *E. coli* (ECOLI), S. *typhimurium* (SALTY), *E. histolytica* (ENTHI), and *M. tuberculosis* (isoform MTCA2_ MYCTU) [11,27,28,29,30]. Conserved amino acids depicted by an asterisk (*), semiconserved ones by (.) or (:).

In order to rationalize the effective catalytic activity of EhiCA, we aligned the amino acid sequence of this protein with that of other β-CAs, such as those from the pathogenic bacteria *Haemophilus influenzae*, *Vibrio cholerae*, *Escherichia coli*, *Salmonella typhimurium*, two isoforms from *Mycobacterium tuberculosis* [11,27,28,29], and the cyanobacterium *Synechocystis* sp. PCC 6803 [30] (Figure 2).

As seen in Figure 2, EhiCA (as all β-CAs investigated to date) has the conserved three zinc(II) ligands, Cys50, His103, and Cys106 (the fourth ligand is presumably a water molecule/hydroxide ion), as well as the catalytic dyad constituted by the pair Asp52–Arg54 (also conserved in all enzymes belonging to this class) [11,12,13,14,15,27,28,29,30], which contributes to the enhancement of the nucleophilicity of the water coordinated to the metal ion. The presence of these conserved amino acids, and all the structural elements connected to them, may explain the catalytic activity of EhiCA reported in this paper (Table 1).

We also investigated the inhibition of EhiCA with a set of inorganic simple and complex anions, as well as small organic molecules known [11,12,13,14,15,27,28,29,30] to interact with CAs, such as diethyl-dithiocarbamate, sulfamide, sulfamic acid, phenyboronic and phenylphosphonic acid, among others (Table 2).

The following observations can be made from the inhibition data shown in Table 2:

(i) The anions which did not show inhibitory activity against EhiCA were fluoride, chloride, and, surprisingly, cyanide and azide, which are highly effective inhibitors of α-CAs such as hCA I and II [32]; pyrodiphosphate and divanadate; perchlorate, tetrafluroborate, hexafluorophosphate, and triflate (which usually do not significantly inhibit any CA [32]); and, again surprisingly, sulfamic acid. All these compounds did not show significant inhibition up to a 100 mM concentration in the assay system.

(ii) The most effective EhiCA inhibitors were sulfamide (which is structurally highly similar to sulfamic acid, except that the pKa of the two compounds is highly different) [33,34] and fluorosulfonate, as well as phenylboronic acid and phenylarsonic acid, which showed K_I_s in the range of 28–86 µM (Table 2). As seen in Table 2, many of these small molecules/anions also act as inhibitors of hCA I and II, but with a rather different efficacy [33].

(iii) Several anions, such as cyanate, selenocyanate, bicarbonate, stannate, tellurate, tetraborate, and *N*,*N*-diethyl-dithiocarbamate were also sub-millimolar EhiCA inhibitors, with K_I_s in the range of 0.28–0.87 mM. Some of these compounds are typical metal complexing agents (cyanate, selenocyanate, *N*,*N*-diethyt-dithiocarbamate), and their propensity to bind the zinc ion in this β-CA explains these inhibitory activities. However, others, (among which are bicarbonate, stannate, tellurate, and tetraborate) show less affinity to act as metal complexing anions [32]. The inhibitory action of bicarbonate, one of the reaction products/substrates of the CA, is particularly interesting, possibly indicating that the enzyme is not acting as a highly efficient bicarbonate dehydratase, but instead that the CO_2_ hydratase activity might be crucial during the life cycle of this protozoan. However, this speculation needs careful validation.

(iv) Many anions acted as low millimolar EhiCA inhibitors. They include iodide, thiocyanate, carbonate, nitrate, nitrite, hydrogensulfide, selenite, perrhenate, perruthenate, peroxydisulfate, trithiocarbonate, and imidosulfonate (K_I_s in the range of 1.7–8.4 mM).

(v) Anions with a less effective inhibitory action against EhiCA were bromide, bisulfite, and sulfate, with K_I_s in the range of 11.5–365.8 mM (Table 1).

## 3. Materials and Methods

### 3.1. Vector Construction

We produced the EhiCA as a recombinant protein in *E. coli*. The DNA sequence was retrieved from UniProt, and modified for recombinant protein production. We provided the sequence of the insert, and the actual construction of the plasmid vector was performed by GeneArt (Invitrogen, Regensburg, Germany). The structure of the insert was specifically modified for production in *E. coli*. The insert was ligated into a modified plasmid vector, pBVboost.

### 3.2. Production of the Protein

The freeze-dried plasmid was prepared, according to manufacturer’s manual. Deep-frozen BL21 Star™ (DE3) cells (Invitrogen, Carlsbad, CA, USA) were slowly melted on ice. Once melted, 25 µL of the cell suspension and 1 µL of the plasmid solution were combined. The suspension was kept on ice for 30 min. Heat shock was performed by submerging the suspension-containing tube into 42 °C water for 30 s, and was then incubated on ice for 2 min. To the tube 125 µL of S.O.C Medium (Invitrogen, Carlsbad, CA, USA) was added, and the tube was incubated for 1 h with constant shaking (200 rpm) at 37 °C. Growth plates (gentamycin-LB medium, ratio 1:1000) were prewarmed at 37 °C for 40 min. Then, 20 µL and 50 µL of suspension was spread on two plates which were incubated overnight at 37 °C. A volume of 5 mL preculture was prepared by inoculating single colonies from growth plates onto an LB medium with gentamycin (ratio 1:1000). It was then incubated overnight at 37 °C with constant shaking (200 rpm). The production was executed according to pO-stat fed batch protocol, which is essentially as described in Määttä et al. [35]. There were some alterations to the previously described protocol: The fermentation medium did not contain glycerol, as the cell line used did not require it. The induction of the culture was performed with 1 mM IPTG, 12 h after starting the fermentation. Temperature was decreased to 25 °C at the time of the induction. Culturing was stopped after 12 h of the induction with the OD 34 (A_600_). The cells were collected by centrifugation, and the wet weight of the cell pellet was 303 g. The fermentation was performed by the Tampere facility of Protein Services (PS). The cell pellet (approximately 35 g) was suspended in 150 mL of binding buffer containing 50 mM Na_2_HPO_4_, 0.5 M NaCl, 50 mM imidazole, and 10% glycerol (pH 8.0), and the suspension was homogenized with an EmulsiFlex-C3 (AVESTIN, Ottawa, Canada) homogenizer. The lysate was centrifuged at 13,000× *g* for 15 min at 4 °C, and the clear supernatant was mixed with HisPur™ Ni-NTA Resin (Thermo Fisher Scientific, Waltham, MA, USA) and bound to the resin for 2 h at room temperature on the magnetic stirrer. Then resin was washed with the binding buffer and collected onto an empty column with an EMD Millipore™ vacuum filtering flask (Merck, Kenilworth, NJ, USA) and filter paper. The protein was eluted from the resin with 50 mM Na_2_HPO_4_, 0.5 M NaCl, 350 mM imidazole, and 10% glycerol (pH 7.0). The protein was re-purified with TALON^®^ Superflow™ cobalt resin (GE Healthcare, Chicago, IL, USA). The eluted protein fractions were diluted with binding buffer (50 mM Na_2_HPO_4_, 0.5 M NaCl, and 10% glycerol pH 8.0), so that the imidazole concentration was under 10 mM. The protein binding and elution was performed as described above. The purity of the protein was determined with gel electrophoresis (SDS-PAGE), and visualized with PageBlue Protein staining solution (Thermo Fisher Scientific, Waltham, MA, USA). Protein fractions were pooled and concentrated with 10 kDa Vivaspin^®^ Turbo 15 centrifugal concentrators (Sartorius™, Göttingen, Germany) at 4000× *g* at 4 °C. Buffer exchange in 50 mM TRIS (pH 7.5) was done using the same centrifugal concentrators. His-tag was cleaved from the purified protein by Thrombin CleanCleave Kit (Sigma-Aldrich, Saint Louis, MO, USA), according to manufacturer’s manual.

### 3.3. CA Activity and Inhibition Measurements

An Sx.18Mv-R Applied Photophysics (Oxford, UK) stopped-flow instrument has been used to assay the catalytic activity of various CA isozymes for the CO_2_ hydration reaction [31]. Phenol red (at a concentration of 0.2 mM) was used as indicator (working at the absorbance maximum of 557 nm). Following the CA-catalyzed CO_2_ hydration reaction, 10 mM Hepes (pH 7.5, for α-CAs) or TRIS (pH 8.3, for β-CAs) as buffers, and 0.1 M NaClO_4_ (for maintaining constant ionic strength), were used for a period of 10 s at 25 °C. The CO_2_ concentrations ranged from 1.7 to 17 mM, for the determination of the kinetic parameters and inhibition constants. For each inhibitor, at least six traces of the initial 5–10% of the reaction were used for determining the initial velocity. The uncatalyzed rates were determined in the same manner, and subtracted from the total observed rates. Stock solutions of inhibitors (10 mM) were prepared in distilled and deionized water, and dilutions of up to 1 µM were done thereafter with the assay buffer. Enzyme and inhibitor solutions were pre-incubated together for 15 min (standard assay at room temperature) prior to assay, in order to allow for the formation of the enzyme–inhibitor complex. The inhibition constants were obtained by non-linear least-squares methods, using PRISM 3 and the Cheng–Prusoff equation [36,37,38].

## 4. Conclusions

In the search for alternative drug targets against anti-protozoan agents, we report the cloning and catalytic activity of a β-CA from *Entamoeba histolytica*, EhiCA, the etiological agent of diarrhea and amebic liver abscesses. This new enzyme has a high catalytic activity for the physiologic CO_2_ hydration reaction, with a k_cat_ of 6.7 × 10^5^ s^−1^ and a k_cat_/K_m_ of 8.9 × 10^7^ M^−1^ × s^−1^. An anion inhibition study of EhiCA with inorganic/organic anions and small molecules was performed, in order to detect interesting leads for effective inhibitors. Fluoride, chloride, cyanide, azide, pyrodiphosphate, perchlorate, tetrafluoroborate, and sulfamic acid did not inhibit the enzyme activity, whereas pseudohalides (cyanate and thiocyanate), bicarbonate, nitrate, nitrite, diethyldithiocarbamate, and many complex inorganic anions showed inhibition in the millimolar range (K_I_s of 0.51–8.4 mM). The best EhiCA inhibitors were fluorosulfonate, sulfamide, phenylboronic acid, and phenylarsonic acid (K_I_s in the range of 28–86 μM). Since β-CAs are not present in vertebrates, the present study may be useful for detecting lead compounds for the design of effective enzyme inhibitors, with potential to develop anti-infectives with alternative mechanisms of action.

## Figures and Tables

**Table 1:** Kinetic parameters for the CO_2_ hydration reaction catalyzed by the human cytosolic isozymes hCA I and II (α-class CAs) at 20 °C and pH 7.5 in 10 mM HEPES buffer and 20 mM Na_2_SO_4_, and the β-CA EhiCA form *E. histolytica* measured at 20 °C, pH 8.3 in 20 mM TRIS buffer and 20 mM NaClO_4_. Inhibition data with the clinically used sulfonamide acetazolamide (5-acetamido-1,3,4-thiadiazole-2-sulfonamide) are also provided [31].

Enzyme	Activity Level	Class	k_cat_ (s^−1^)	K_m_ (mM)	k_cat_/K_m_ (M^−1^ × s^−1^)	K_I_ (Acetazolamide) (nM)
hCA I	moderate	α	2.0 × 10^5^	4.0	5.0 × 10^7^	250
hCA II	very high	α	1.4 × 10^6^	9.3	1.5 × 10^8^	2
EhiCA	high	β	(6.7 ± 0.2) × 10^5^	7.5 ± 0.08	(8.9 ± 0.1) × 10^7^	509

**Table 2:** Inhibition constants of anionic inhibitors against the α-CA isoforms hCA II and hCA I, as well as the β-class protozoan enzyme EhiCA, for the CO_2_ hydration reaction at 20 °C [31].

Inhibitor ^§^	K_I_ [mM] ^#^		
	hCA II	hCA I	EhiCA
F^−^	>300	>300	>100
Cl^−^	200	6.0	>100
Br^−^	63	4.1	36.8
I^−^	26	0.3	7.4
CNO^−^	0.03	0.0007	0.77
SCN^−^	1.6	0.2	7.9
CN^−^	0.02	0.0005	>100
N_3_^−^	1.51	0.0012	>100
HCO_3_^−^	85	12	0.28
CO_3_^2−^	73	15	2.4
NO_3_^−^	35	7.0	3.6
NO_2_^−^	63	8.4	1.7
HS^−^	0.04	0.0006	6.9
HSO_3_^−^	89	18	11.5
SO_4_^2−^	>200	63	21.6
SnO_3_^2−^	0.83	0.57	0.51
SeO_4_^2−^	112	118	6.0
TeO_4_^2−^	0.92	0.66	0.61
P_2_O_7_^4−^	48.50	25.8	>100
V_2_O_7_^4−^	0.57	0.54	>100
B_4_O_7_^2−^	0.95	0.64	0.29
ReO_4_^−^	0.75	0.11	7.1
RuO_4_^−^	0.69	0.10	7.0
S_2_O_8_^2−^	0.084	0.11	8.4
SeCN^−^	0.086	0.085	0.87
CS_3_^2−^	0.0088	0.0087	6.0
Et_2_NCS_2_^−^	3.1	0.00079	0.51
ClO_4_^−^	>200	>200	>100
BF_4_^−^	>200	>200	>100
FSO_3_^−^	0.46	0.79	0.086
PF_6_^−^	>200	>200	>100
CF_3_SO_3_^−^	>200	>200	>100
NH(SO_3_)_2_^2−^	0.76	0.31	2.2
H_2_NSO_2_NH_2_	1.13	0.31	0.028
H_2_NSO_3_H	0.39	0.021	>100
Ph-B(OH)_2_	23.1	38.6	0.047
Ph-AsO_3_H_2_	49.2	31.7	0.038

^§^ As sodium salt; ^#^ Errors were in the range of 3–5% of the reported values, from three different assays.
